# A new trigonometric modification of the Weibull distribution: Control chart and applications in quality control

**DOI:** 10.1371/journal.pone.0286593

**Published:** 2023-07-12

**Authors:** Mohammed Ahmed Alomair, Zubair Ahmad, Gadde Srinivasa Rao, Hazem Al-Mofleh, Saima Khan Khosa, Abdulaziz Saud Al Naim

**Affiliations:** 1 Department of Quantitative Methods, School of Business, King Faisal University, Al-Ahsa, Saudi Arabia; 2 Department of Statistics, Quad-i-Azam University, Islamabad, Pakistan; 3 Department of Mathematics and Statistics, University of Dodoma, Dodoma, Tanzania; 4 Department of Mathematics, Tafila Technical University, Tafila, Jordan; 5 Department of Mathematics and Statistics University of Saskatchewan, Saskatoon, SK, Canada; 6 Accounting Department, Business School, King Faisal University, Al-Ahsa, Saudi Arabia; University of Bradford, UNITED KINGDOM

## Abstract

In the most recent era, the extensions of the probability models via trigonometry methods have received great attention. This paper also offers a novel trigonometric version of the Weibull model called a type-I cosine exponentiated Weibull (for short “TICE-Weibull”) distribution. The identifiability properties for all three parameters of the TICE-Weibull model are derived. The estimators of the TICE-Weibull model are derived by implementing the maximum likelihood approach. To demonstrate the effectiveness of the TICE-Weibull model, two applications from real-world phenomena are analyzed. In addition, the proposed statistical model is established for an attribute control chart based on a time-truncated life test. The advantage of the developed charts is examined based on the average run length (ARL). The necessary tables of shift sizes and various sample sizes are offered for numerous values of the distribution parameters, as well as specified ARL and shift constants. Some numerical examples are discussed for various scheme parameters to study the performance of the new TICE-Weibull attribute control charts. According to our search and a brief study of the statistical literature, there is no published work on the development of a control chart using new probability models that are introduced using the cosine function. This is the key motivation of this work, which fills this amazing and interesting research gap.

## 1 Introduction

The two parameters (*α* > 0, *δ* > 0) Weibull model is one of the most famous and important probability models that can best describe the failure behavior of the system/components during its lifetime. It is defined on $R^{+}$ and has been implemented for analyzing lifetime phenomena in engineering and other close connected fields (Pham and Lai [1], Almalki and Nadarajah [2]).

To update the flexibility, characteristics, and data fitting of the Weibull model, numerous new versions (extended forms) of the Weibull model has been introduced, studied, and recommended; see Basheer [3], Shakhatreh et al. [4], Mazucheli et al. [5], Nassar et al. [6], Elgohari and Yousof [7], Strzelecki [8], Sindhu and Atangana [9], Vanem Fazeres-Ferradosa [10], Ahmad et al. [11], and Zhao et al. [12].

The up-gradation of the Weibull model has been done by implementing numerous approaches; see Ahmad et al. [13]. However, the literature on distribution theory has lack probability distributions that are based on trigonometric functions; most of these modifications are based on algebraic functions. The increased interest in data analysis, modeling, predicting, and directional data analysis, in particular, motivates researchers to look for the development of new approaches based on trigonometric functions. Therefore, in the recent literature about the development of new updated versions of the Weibull model, researchers have focused on implementing trigonometric functions; see Chesneau et al. [14].

The one explored by Souza et al. [15] provides a modern alternative based on a trigonometric function. They introduced a new class of distributions called a sin-*G* class of probability models with cumulative distribution function (CDF) *F*(*x*; ***ζ***) given by

$$\begin{array}{c} F(x;\zeta )=\text{sin}[\frac{\pi }{2}G(x;\zeta )], \end{array}$$

where *G*(*x*; ***ζ***) is CDF of the baseline random variable depending on the vector of parameters ***ζ***.

Silveira et al. [16] introduced the normal-tangent-*G* (NT-*G*) class of distributions for studying new statistical models. The CDF *F*(*x*; ***ζ***) of the NT-*G* distributions is given by

$$\begin{array}{c} F(x;\zeta )=\Phi (\text{tan}[\pi (G(x;\zeta )-\frac{1}{2})]). \end{array}$$

Another contribution work towards the trigonometric family of distributions is due to Jamal et al. [17]. They introduced a generalized version of the sin-*G* method called the transformed sin-*G* (TS-*G*) family of distributions. The CDF *F*(*x*; *η*, ***ζ***) of the TS-*G* family is

$$\begin{array}{c} F(x;\eta ,\zeta )=\text{sin}[\frac{\pi }{2}G(x;\zeta )]-\eta \frac{\pi }{2}G(x;\zeta )\text{cos}[\frac{\pi }{2}G(x;\zeta )], \end{array}$$

where *η* = ∈ [0, 1].

Nanga et al. [18] introduced the tangent Topp-Leone (TTL) family of distributions having CDF *F*(*x*; *β*, ***ζ***) given by

$$\begin{array}{c} F(x;\beta ,\zeta )=\text{tan}[\frac{\pi }{4}{\left(1-{\left[1-G(x;\zeta )\right]}^{2}\right)}^{\beta }], \end{array}$$

where *β* > 0.

From the above literature, we can see that researchers are turning their attention to introducing new probability distributions using trigonometric functions. However, based on our deep study of the literature, there is no published work about the construction of new control charts using new probability distributions that are based on the cosine function. In this paper, first, we use a trigonometric function to derive a new form of the Weibull distribution. Then, using the proposed model, we construct a new control chart. This is a key motivation for this paper.

The new form of the Weibull model is introduced by using the trigonometric function called Type-I cosine exponentiated-*X* (TICE-*X*) family. Suppose *X* has the TICE-*X* family, if its CDF is given by

$$\begin{array}{c} F(x;\theta ,\zeta )=\frac{e^{\text{cos}(\frac{\pi }{2}\{1-{\left[G(x;\zeta )\right]}^{\theta }\})}-1}{e-1},\;\;\;\;\;\;\;\;\theta >0,x\in R, \end{array}$$ (1)

with PDF given by

$$\begin{array}{c} f(x;\theta ,\zeta )=\frac{\theta \pi g(x;\zeta ){\left[G(x;\zeta )\right]}^{\theta -1}\text{sin}(\frac{\pi }{2}\{1-{\left[G(x;\zeta )\right]}^{\theta }\})}{2(e-1)}e^{\text{cos}(\frac{\pi }{2}\{1-{\left[G(x;\zeta )\right]}^{\theta }\})}, \end{array}$$ (2)

where *g*(*x*; ***ζ***) is PDF of the baseline random variable depending on the vector of parameters ***ζ***. With respect to *F*(*x*; *θ*, ***ζ***) and *f*(*x*; *θ*, ***ζ***), the hazard function *h*(*x*; *θ*, ***ζ***), cumulative HF *h*(*x*; *θ*, ***ζ***), and survival function (SF) *S*(*x*; *θ*, ***ζ***) of the TICE-*X* distributions are given by

$$\begin{array}{c} h\left(x;\theta ,\zeta \right)=\frac{\theta \pi g\left(x;\zeta \right){\left[G\left(x;\zeta \right)\right]}^{\theta -1}\text{sin}\left(\frac{\pi }{2}\left\{1-{\left[G\left(x;\zeta \right)\right]}^{\theta }\right\}\right)}{\left[e-e^{\text{cos}\left(\frac{\pi }{2}\left\{1-{\left[G\left(x;\zeta \right)\right]}^{\theta }\right\}\right)}\right]}e^{\text{cos}\left(\frac{\pi }{2}\left\{1-{\left[G\left(x;\zeta \right)\right]}^{\theta }\right\}\right)},\\ H\left(x;\theta ,\zeta \right)=-\text{log}\left[\frac{e-e^{\text{cos}\left(\frac{\pi }{2}\left\{1-{\left[G\left(x;\zeta \right)\right]}^{\theta }\right\}\right)}}{e-1}\right], \end{array}$$

and

$$\begin{array}{c} S(x;\theta ,\zeta )=\frac{e-e^{\text{cos}(\frac{\pi }{2}\{1-{\left[G(x;\zeta )\right]}^{\theta }\})}}{e-1}, \end{array}$$

respectively.

In this paper, we implement the method presented in Eq (1) to define/introduce a new trigonometric-Weibull (TICE-Weibull) distribution. Section 2 offers the definition of the basic functions of the TICE-Weibull distribution. The plots for the PDF of the TICE-Weibull distribution are also presented in Section 2. Some distributional properties of the TICE-Weibull distribution are obtained in Section 3. A simulation study in Section 4 is provided to evaluate the performances of the estimators of the TICE-Weibull distribution using two well-known statistical criteria called (*i*) the average bias, and (*ii*) the average mean square error. The applicability of the TICE-Weibull distribution is shown by considering two real-life applications in Section 5. An attribute control chart based on truncated life tests is given in Section 6 and in Section 7, the final remarks are provided.

## 2 Special case

This section offers a special case of the TICE-*X* family of distributions, namely, a TICE-Weibull distribution. The TICE-Weibull can be considered an updated form of the Weibull model with CDF *G*(*x*; ***ζ***) given by

$$\begin{array}{c} G(x;\zeta )=1-e^{-\delta x^{\alpha }},\;\;\;\;\;\;\;\;x\geq 0,\alpha ,\delta >0, \end{array}$$ (3)

and PDF *g*(*x*; ***ζ***) given by

$$\begin{array}{c} g(x;\zeta )=\alpha \delta x^{\alpha -1}e^{-\delta x^{\alpha }},\;\;\;\;\;\;\;\;x>0, \end{array}$$ (4)

where ***ζ*** = (*α*, *δ*)^⊤^.

The CDF *F*(*x*; *θ*, ***ζ***) of the TICE-Weibull model is obtained using Eq (3) in Eq (1). So, the CDF of the TICE-Weibull model has the following form

$$\begin{array}{c} F(x;\theta ,\zeta )=\frac{e^{\text{cos}(\frac{\pi }{2}\{1-{\left[1-e^{-\delta x^{\alpha }}\right]}^{\theta }\})}-1}{e-1},\;\;\;\;\;\;\;\;x\geq 0. \end{array}$$ (5)

Using Eqs (3) and (4) in Eq (2), we get the PDF *F*(*x*; *θ*, ***ζ***) of the TICE-Weibull model, given by

$$\begin{array}{cc} f(x;\theta ,\zeta ) & =\frac{\pi \theta \alpha \delta x^{\alpha -1}e^{-\delta x^{\alpha }}{\left[1-e^{-\delta x^{\alpha }}\right]}^{\theta -1}\text{sin}(\frac{\pi }{2}\{1-{\left[1-e^{-\delta x^{\alpha }}\right]}^{\theta }\})}{2(e-1)}\\ & \times e^{\text{cos}(\frac{\pi }{2}\{1-{\left[1-e^{-\delta x^{\alpha }}\right]}^{\theta }\})}. \end{array}$$ (6)

**Fig 1:**
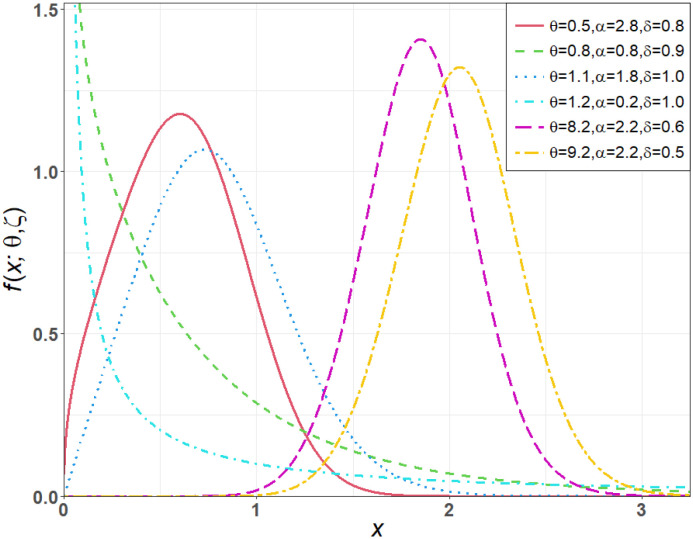
Visual behavior of *f*(*x*; *θ*, ζ) of the TICE-Weibull model.

Different behaviors of the PDF *f*(*x*; *θ*, ***ζ***) of the TICE-Weibull model are visually illustrated in Fig 1. Fig 1 shows that *f*(*x*; *θ*, ***ζ***) of the TICE-Weibull model has four possible shapes (behaviors). These shapes include (i) decreasing shape (*θ* = 0.8, *α* = 0.8, *δ* = 0.9 and *θ* = 1.2, *α* = 0.2, *δ* = 1.0), (ii) symmetrical shape (*θ* = 8.2, *α* = 2.2, *δ* = 0.6), (iii) right-skewed shape (*θ* = 0.5, *α* = 2.8, *δ* = 0.8 and *θ* = 1.1, *α* = 1.8, *δ* = 1.0) and (iv) left-skewed shape (*θ* = 9.2, *α* = 2.2, *δ* = 0.5).

## 3 Mathematical properties

This section of the paper deals with the computation of some mathematical properties of the TICE-Weibull distribution. These properties include the quantile function (QF), identifiability property (IP), and *m*^*th*^ moment.

### 3.1 The quantile function

The quantile of the TICE-Weibull distribution, say *x*_*q*_, is obtained by solving the following equation for *x*

$$\begin{array}{c} F(x;\theta ,\zeta )=q, \end{array}$$ (7)

where 0 < *q* < 1. Using Eq (5) in Eq (7), we get

$$\begin{array}{c} \frac{e^{\text{cos}(\frac{\pi }{2}\{1-{\left[1-e^{-\delta x_{q}^{\alpha }}\right]}^{\theta }\})}-1}{e-1}=q. \end{array}$$ (8)

On solving Eq (8), we get the QF of the TICE-Weibull distribution, which is given by

$$x_{q}={\left(-\frac{1}{\delta }\;\text{log}\left[1-{\left\{1-\frac{2}{\pi }{\text{cos}}^{-1}\left(\text{log}\left[q\left(e-1\right)+1\right]\right)\right\}}^{1/\theta }\right]\right)}^{1/\alpha }.$$ (9)

### 3.2 The IP of the TICE-Weibull model

In this subsection, we prove the IP of the TICE-Weibull distribution using the parameters *α*, *δ*, and *θ*.

#### 3.2.1 The IP of the TICE-Weibull model using *α*

Suppose *α*_1_ and *α*_2_ are the model parameters of the TICE-Weibull model with CDFs

$$\begin{array}{c} F(x;\theta ,\zeta_{1})=\frac{e^{\text{cos}(\frac{\pi }{2}\{1-{\left[1-e^{-\delta x^{\alpha_{1}}}\right]}^{\theta }\})}-1}{e-1}, \end{array}$$

and

$$\begin{array}{c} F(x;\theta ,\zeta_{2})=\frac{e^{\text{cos}(\frac{\pi }{2}\{1-{\left[1-e^{-\delta x^{\alpha_{2}}}\right]}^{\theta }\})}-1}{e-1}, \end{array}$$

where ***ζ***_**1**_ = (*α*_1_, *δ*)^⊤^ and ***ζ***_**2**_ = (*α*_2_, *δ*)^⊤^, respectively. The parameter *α* will be identifiable, if *F*(*x*; *θ*, ***ζ***_**1**_) = *F*(*x*; *θ*, ***ζ***_**2**_), we can prove *α*_1_ = *α*_2_.

**Proof:** We start with *F*(*x*; *θ*, ***ζ***_**1**_) = *F*(*x*; *θ*, ***ζ***_**2**_), this follows

$$\begin{array}{c} \frac{e^{\text{cos}(\frac{\pi }{2}\{1-{\left[1-e^{-\delta x^{\alpha_{1}}}\right]}^{\theta }\})}-1}{e-1}=\frac{e^{\text{cos}(\frac{\pi }{2}\{1-{\left[1-e^{-\delta x^{\alpha_{2}}}\right]}^{\theta }\})}-1}{e-1}, \end{array}$$

$$\begin{array}{c} e^{\text{cos}(\frac{\pi }{2}\{1-{\left[1-e^{-\delta x^{\alpha_{1}}}\right]}^{\theta }\})}=e^{\text{cos}(\frac{\pi }{2}\{1-{\left[1-e^{-\delta x^{\alpha_{2}}}\right]}^{\theta }\})}, \end{array}$$

$$\begin{array}{c} \text{cos}(\frac{\pi }{2}\{1-{\left[1-e^{-\delta x^{\alpha_{1}}}\right]}^{\theta }\})=\text{cos}(\frac{\pi }{2}\{1-{\left[1-e^{-\delta x^{\alpha_{2}}}\right]}^{\theta }\}). \end{array}$$

Now, since

$$\begin{array}{c} 0<1-e^{-\delta x^{\alpha_{1}}}<1\;\;\text{and}\;\;0<1-e^{-\delta x^{\alpha_{2}}}<1. \end{array}$$

Then

$$\begin{array}{c} 0<\frac{\pi }{2}\{1-{\left[1-e^{-\delta x^{\alpha_{1}}}\right]}^{\theta }\}<\frac{\pi }{2}\;\;\text{and}\;\;0<\frac{\pi }{2}\{1-{\left[1-e^{-\delta x^{\alpha_{2}}}\right]}^{\theta }\}<\frac{\pi }{2}. \end{array}$$

Thus, the domain of

$$\begin{array}{c} \text{cos}(\frac{\pi }{2}\{1-{\left[1-e^{-\delta x^{\alpha_{1}}}\right]}^{\theta }\})\;\;\text{and}\;\;\text{cos}(\frac{\pi }{2}\{1-{\left[1-e^{-\delta x^{\alpha_{2}}}\right]}^{\theta }\}) \end{array}$$

is $\left(0,\frac{\pi }{2}\right)$, and the cosine function on this interval is an one-to-one function (i.e. if cos(*γ*_1_) = cos(*γ*_2_) implies *γ*_1_ = *γ*_2_). Therefore,

$$\begin{array}{c} \frac{\pi }{2}\{1-{\left[1-e^{-\delta x^{\alpha_{1}}}\right]}^{\theta }\}=\frac{\pi }{2}\{1-{\left[1-e^{-\delta x^{\alpha_{2}}}\right]}^{\theta }\}, \end{array}$$

$$\begin{array}{c} {\left[1-e^{-\delta x^{\alpha_{1}}}\right]}^{\theta }={\left[1-e^{-\delta x^{\alpha_{2}}}\right]}^{\theta }, \end{array}$$

$$\begin{array}{c} e^{-\delta x^{\alpha_{1}}}=e^{-\delta x^{\alpha_{2}}}, \end{array}$$

$$\begin{array}{c} x^{\alpha_{1}}=x^{\alpha_{2}}, \end{array}$$

$$\begin{array}{c} \alpha_{1}=\alpha_{2}. \end{array}$$

#### 3.2.2 The IP of the TICE-Weibull model using *δ*

Suppose *δ*_1_ and *δ*_2_ are the model parameters of the TICE-Weibull model with CDFs

$$\begin{array}{c} F(x;\theta ,\zeta_{1})=\frac{e^{\text{cos}(\frac{\pi }{2}\{1-{\left[1-e^{-\delta_{1}x^{\alpha }}\right]}^{\theta }\})}-1}{e-1}, \end{array}$$

and

$$\begin{array}{c} F(x;\theta ,\zeta_{2})=\frac{e^{\text{cos}(\frac{\pi }{2}\{1-{\left[1-e^{-\delta_{2}x^{\alpha }}\right]}^{\theta }\})}-1}{e-1}, \end{array}$$

where ***ζ***_**1**_ = (*α*_1_, *δ*)^⊤^ and ***ζ***_**2**_ = (*α*_2_, *δ*)^⊤^, respectively. The parameter *δ* will be identifiable, if *F*(*x*; *θ*, ***ζ***_**1**_) = *F*(*x*; *θ*, ***ζ***_**2**_), we can prove *δ*_1_ = *δ*_2_.

**Proof:** We start with *F*(*x*; *θ*, ***ζ***_**1**_) = *F*(*x*; *θ*, ***ζ***_**2**_), this follows

$$\begin{array}{c} \frac{e^{\text{cos}(\frac{\pi }{2}\{1-{\left[1-e^{-\delta_{1}x^{\alpha }}\right]}^{\theta }\})}-1}{e-1}=\frac{e^{\text{cos}(\frac{\pi }{2}\{1-{\left[1-e^{-\delta_{2}x^{\alpha }}\right]}^{\theta }\})}-1}{e-1}, \end{array}$$

$$\begin{array}{c} e^{\text{cos}(\frac{\pi }{2}\{1-{\left[1-e^{-\delta_{1}x^{\alpha }}\right]}^{\theta }\})}=e^{\text{cos}(\frac{\pi }{2}\{1-{\left[1-e^{-\delta_{2}x^{\alpha }}\right]}^{\theta }\})}, \end{array}$$

$$\begin{array}{c} \text{cos}(\frac{\pi }{2}\{1-{\left[1-e^{-\delta_{1}x^{\alpha }}\right]}^{\theta }\})=\text{cos}(\frac{\pi }{2}\{1-{\left[1-e^{-\delta_{2}x^{\alpha }}\right]}^{\theta }\}). \end{array}$$

Now, since

$$\begin{array}{c} 0<1-e^{-\delta_{1}x^{\alpha }}<1\;\;\text{and}\;\;0<1-e^{-\delta_{2}x^{\alpha }}<1. \end{array}$$

Then

$$\begin{array}{c} 0<\frac{\pi }{2}\{1-{\left[1-e^{-\delta_{1}x^{\alpha }}\right]}^{\theta }\}<\frac{\pi }{2}\;\;\text{and}\;\;0<\frac{\pi }{2}\{1-{\left[1-e^{-\delta_{2}x^{\alpha }}\right]}^{\theta }\}<\frac{\pi }{2}. \end{array}$$

Thus, the domain of

$$\begin{array}{c} \text{cos}(\frac{\pi }{2}\{1-{\left[1-e^{-\delta_{1}x^{\alpha }}\right]}^{\theta }\})\;\;\text{and}\;\;\text{cos}(\frac{\pi }{2}\{1-{\left[1-e^{-\delta_{2}x^{\alpha }}\right]}^{\theta }\}) \end{array}$$

is $\left(0,\frac{\pi }{2}\right)$, and the cosine function on this interval is an one-to-one function (i.e. if cos(*γ*_1_) = cos(*γ*_2_) implies *γ*_1_ = *γ*_2_). Therefore,

$$\begin{array}{c} \frac{\pi }{2}\{1-{\left[1-e^{-\delta_{1}x^{\alpha }}\right]}^{\theta }\}=\frac{\pi }{2}\{1-{\left[1-e^{-\delta_{2}x^{\alpha }}\right]}^{\theta }\}, \end{array}$$

$$\begin{array}{c} {\left[1-e^{-\delta_{1}x^{\alpha }}\right]}^{\theta }={\left[1-e^{-\delta_{2}x^{\alpha }}\right]}^{\theta }, \end{array}$$

$$\begin{array}{c} e^{-\delta_{1}x^{\alpha }}=e^{-\delta_{2}x^{\alpha }}, \end{array}$$

$$\begin{array}{c} \delta_{1}x^{\alpha }=\delta_{2}x^{\alpha }, \end{array}$$

$$\begin{array}{c} \delta_{1}=\delta_{2}. \end{array}$$

#### 3.2.3 The IP of the TICE-Weibull model using *θ*

Suppose *θ*_1_ and *θ*_2_ are the model parameters of the TICE-Weibull model with CDFs

$$\begin{array}{c} F(x;\theta_{1},\zeta )=\frac{e^{\text{cos}(\frac{\pi }{2}\{1-{\left[1-e^{-\delta x^{\alpha }}\right]}^{\theta_{1}}\})}-1}{e-1}, \end{array}$$

and

$$\begin{array}{c} F(x;\theta_{2},\zeta )=\frac{e^{\text{cos}(\frac{\pi }{2}\{1-{\left[1-e^{-\delta x^{\alpha }}\right]}^{\theta_{2}}\})}-1}{e-1}, \end{array}$$

respectively. The parameter *θ* will be identifiable, if *F*(*x*; *θ*_1_, ***ζ***) = *F*(*x*; *θ*_2_, ***ζ***), we can prove *θ*_1_ = *θ*_2_.

**Proof:** We start with *F*(*x*; *θ*_1_, ***ζ***) = *F*(*x*; *θ*_2_, ***ζ***), this follows

$$\begin{array}{c} \frac{e^{\text{cos}(\frac{\pi }{2}\{1-{\left[1-e^{-\delta x^{\alpha }}\right]}^{\theta_{1}}\})}-1}{e-1}=\frac{e^{\text{cos}(\frac{\pi }{2}\{1-{\left[1-e^{-\delta x^{\alpha }}\right]}^{\theta_{2}}\})}-1}{e-1}, \end{array}$$

$$\begin{array}{c} e^{\text{cos}(\frac{\pi }{2}\{1-{\left[1-e^{-\delta x^{\alpha }}\right]}^{\theta_{1}}\})}=e^{\text{cos}(\frac{\pi }{2}\{1-{\left[1-e^{-\delta x^{\alpha }}\right]}^{\theta_{2}}\})}, \end{array}$$

$$\begin{array}{c} \text{cos}(\frac{\pi }{2}\{1-{\left[1-e^{-\delta x^{\alpha }}\right]}^{\theta_{1}}\})=\text{cos}(\frac{\pi }{2}\{1-{\left[1-e^{-\delta x^{\alpha }}\right]}^{\theta_{2}}\}). \end{array}$$

Now, since

$$\begin{array}{c} 0<1-e^{-\delta x^{\alpha }}<1. \end{array}$$

Then

$$\begin{array}{c} 0<\frac{\pi }{2}\{1-{\left[1-e^{-\delta x^{\alpha }}\right]}^{\theta_{1}}\}<\frac{\pi }{2}\;\;\text{and}\;\;0<\frac{\pi }{2}\{1-{\left[1-e^{-\delta x^{\alpha }}\right]}^{\theta_{2}}\}<\frac{\pi }{2}. \end{array}$$

Thus, the domain of

$$\begin{array}{c} \text{cos}(\frac{\pi }{2}\{1-{\left[1-e^{-\delta x^{\alpha }}\right]}^{\theta_{1}}\})\;\;\text{and}\;\;\text{cos}(\frac{\pi }{2}\{1-{\left[1-e^{-\delta x^{\alpha }}\right]}^{\theta_{2}}\}), \end{array}$$

is $\left(0,\frac{\pi }{2}\right)$, and the cosine function on this interval is an one-to-one function (i.e. if cos(*γ*_1_) = cos(*γ*_2_) implies *γ*_1_ = *γ*_2_). Therefore,

$$\begin{array}{c} \frac{\pi }{2}\{1-{\left[1-e^{-\delta x^{\alpha }}\right]}^{\theta_{1}}\}=\frac{\pi }{2}\{1-{\left[1-e^{-\delta x^{\alpha }}\right]}^{\theta_{2}}\}, \end{array}$$

$$\begin{array}{c} {\left[1-e^{-\delta x^{\alpha }}\right]}^{\theta_{1}}={\left[1-e^{-\delta x^{\alpha }}\right]}^{\theta_{2}}, \end{array}$$

$$\begin{array}{c} \theta_{1}\;\text{log}\left[1-e^{-\delta x^{\alpha }}\right]=\theta_{2}\;\text{log}\left[1-e^{-\delta x^{\alpha }}\right], \end{array}$$

$$\begin{array}{c} \theta_{1}=\theta_{2}. \end{array}$$

### 3.3 The *m*^*th*^ moment

Here, we obtain the *m*^*th*^ moment of the TICE-Weibull model as follows

$$\begin{array}{c} \mu_{m}^{\prime }=\int_{\Omega }x^{m}f(x;\theta ,\zeta )dx. \end{array}$$ (10)

Using Eq (2) in Eq (10), we have

$$\begin{array}{c} \mu_{m}^{\prime }=\int_{\Omega }x^{m}\frac{\theta \pi g(x;\zeta ){\left[G(x;\zeta )\right]}^{\theta -1}\text{sin}(\frac{\pi }{2}\{1-{\left[G(x;\zeta )\right]}^{\theta }\})}{2(e-1)}e^{\text{cos}(\frac{\pi }{2}\{1-{\left[G(x;\zeta )\right]}^{\theta }\})}dx, \end{array}$$

$$\begin{array}{cc} \mu_{m}^{\prime } & =\sum_{i=1}^{m}\frac{1}{i!}\int_{\Omega }x^{m}\frac{\theta \pi g(x;\zeta ){\left[G(x;\zeta )\right]}^{\theta -1}\text{sin}(\frac{\pi }{2}\{1-{\left[G(x;\zeta )\right]}^{\theta }\})}{2(e-1)}\\ & \times {\left[\text{cos}(\frac{\pi }{2}\{1-{\left[G(x;\zeta )\right]}^{\theta }\})\right]}^{i}dx, \end{array}$$

$$\begin{array}{cc} \mu_{m}^{\prime } & =\sum_{i=1}^{m}\frac{1}{i!}\int_{\Omega }x^{m}\frac{\theta \pi g(x;\zeta ){\left[G(x;\zeta )\right]}^{\theta -1}[1-(\frac{\pi }{2}\{1-{\left[G(x;\zeta )\right]}^{\theta }\})]}{2(e-1)}\\ & \times {\left[\text{cos}(\frac{\pi }{2}\{1-{\left[G(x;\zeta )\right]}^{\theta }\})\right]}^{i}dx, \end{array}$$

$$\begin{array}{cc} \mu_{m}^{\prime } & =\sum_{i=1}^{m}\frac{1}{i!}\int_{\Omega }x^{m}\frac{\theta \pi g(x;\zeta ){\left[G(x;\zeta )\right]}^{\theta -1}}{2(e-1)}{\left[\text{cos}(\frac{\pi }{2}\{1-{\left[G(x;\zeta )\right]}^{\theta }\})\right]}^{i}dx\\ & -\sum_{i=1}^{m}\frac{1}{i!}\int_{\Omega }x^{m}\frac{\theta \pi g(x;\zeta ){\left[G(x;\zeta )\right]}^{\theta -1}}{2(e-1)}{\left[\text{cos}(\frac{\pi }{2}\{1-{\left[G(x;\zeta )\right]}^{\theta }\})\right]}^{i+1}dx, \end{array}$$

$$\begin{array}{cc} \mu_{m}^{\prime } & =\sum_{i=1}^{m}\frac{\alpha \delta \theta \pi }{i!2(e-1)}[A(x;\theta ,\zeta )+B(x;\theta ,\zeta )], \end{array}$$

where *A*(*x*; *θ*, ***ζ***) and *B*(*x*; *θ*, ***ζ***) are given by

$$\begin{array}{c} A(x;\theta ,\zeta )=\int_{0}^{\infty }x^{m+\alpha -1}e^{-\delta x^{\alpha }}{\left[1-e^{-\delta x^{\alpha }}\right]}^{\theta -1}{\left[\text{cos}(\frac{\pi }{2}\{1-{\left[1-e^{-\delta x^{\alpha }}\right]}^{\theta }\})\right]}^{i}dx, \end{array}$$

and

$$\begin{array}{c} B(x;\theta ,\zeta )=\int_{0}^{\infty }x^{m+\alpha -1}e^{-\delta x^{\alpha }}{\left[1-e^{-\delta x^{\alpha }}\right]}^{\theta -1}{\left[\text{cos}(\frac{\pi }{2}\{1-{\left[1-e^{-\delta x^{\alpha }}\right]}^{\theta }\})\right]}^{i+1}dx, \end{array}$$

respectively.

## 4 Estimation and simulation

This section has two aims. The very first aim concerns the derivation of the maximum likelihood estimators (MLEs) $\left({\hat{\theta }}_{MLE},{\hat{\zeta }}_{MLE}\right)$ of the parameters (*θ*, ***ζ***) of the TICE-Weibull distribution. The second aim of this section is to demonstrate the performances of ${\hat{\theta }}_{MLE}$ and ${\hat{\zeta }}_{MLE}.$

### 4.1 Estimation

Consider a sample of size *k*, say *x*_1_, *x*_2_, …., *x*_*k*_, taken randomly from the TICE-Weibull model with PDF in Eq (2). The likelihood function (LH) λ(*θ*, ***ζ***|*x*_1_, *x*_2_, …., *x*_*k*_) corresponding to Eq (2), is given

$$\begin{array}{c} \lambda (\theta ,\zeta |x_{1},x_{2},....,x_{k})=\prod_{v=1}^{k}\;f(x;\theta ,\zeta ). \end{array}$$ (11)

Using Eq (2) in Eq (11), we get

$$\begin{array}{cc} \lambda (\theta ,\zeta |x_{1},x_{2},....,x_{k}) & =\prod_{v=1}^{k}\frac{\theta \pi g(x;\zeta ){\left[G(x;\zeta )\right]}^{\theta -1}\text{sin}(\frac{\pi }{2}\{1-{\left[G(x;\zeta )\right]}^{\theta }\})}{2(e-1)}\\ & \times e^{\text{cos}(\frac{\pi }{2}\{1-{\left[G(x;\zeta )\right]}^{\theta }\})}. \end{array}$$ (12)

Using Eqs (3) and (4) in Eq (12), we get

$$\begin{array}{cc} \lambda (\theta ,\zeta |x_{1},x_{2},....,x_{k}) & =\prod_{v=1}^{k}\frac{\pi \theta \alpha \delta x^{\alpha -1}e^{-\delta x^{\alpha }}\text{sin}(\frac{\pi }{2}\{1-{\left[1-e^{-\delta x^{\alpha }}\right]}^{\theta }\})}{2(e-1)}\\ & \times {\left[1-e^{-\delta x^{\alpha }}\right]}^{\theta -1}e^{\text{cos}(\frac{\pi }{2}\{1-{\left[1-e^{-\delta x^{\alpha }}\right]}^{\theta }\})}. \end{array}$$ (13)

Corresponding to λ(*θ*, ***ζ***|*x*_1_, *x*_2_, …., *x*_*k*_) in Eq (13), the log LF, say *ξ*(*x*_1_, *x*_2_, …., *x*_*k*_|*θ*, ***ζ***), is given by

$$\begin{array}{cc} \xi (x_{1},x_{2},....,x_{k}|\theta ,\zeta ) & =k\;\text{log}\;\pi +k\;\text{log}\;\theta +k\;\text{log}\;\alpha +k\;\text{log}\;\delta +(\alpha -1)\sum_{i=1}^{k}\;\text{log}\;x_{i}\\ & +\sum_{i=1}^{k}\text{cos}(\frac{\pi }{2}\{1-{\left[1-e^{-\delta x_{i}^{\alpha }}\right]}^{\theta }\})-k\;\text{log}\;\left[2(e-1)\right]\\ & -\delta \sum_{i=1}^{k}x_{i}^{\alpha }+\sum_{i=1}^{k}\;\text{log}\;\text{sin}(\frac{\pi }{2}\{1-{\left[1-e^{-\delta x_{i}^{\alpha }}\right]}^{\theta }\})\\ & +(\theta -1)\sum_{i=1}^{k}\;\text{log}\;[1-e^{-\delta x_{i}^{\alpha }}]. \end{array}$$ (14)

Corresponding to *α*, *δ*, and *θ*, the derivatives of the expression *ξ*(*x*_1_, *x*_2_, …., *x*_*k*_|*θ*, ***ζ***) in Eq (14) are, respectively, given by

$$\begin{array}{cc} \frac{\partial }{\partial \alpha }\xi (x_{1},x_{2},....,x_{k}|\theta ,\zeta ) & =+\frac{\theta \pi }{2}\sum_{i=1}^{k}\text{sin}(\frac{\pi }{2}\{1-{\left[A(x_{i})\right]}^{\theta }\})\left[\text{log}\;x_{i}\right]x_{i}^{\alpha }e^{-\delta x^{\alpha }}{\left[A(x_{i})\right]}^{\theta -1}\\ & -\frac{\theta \pi }{2}\sum_{i=1}^{k}\frac{\text{cos}(\frac{\pi }{2}\{1-{\left[A(x_{i})\right]}^{\theta }\})\left[\text{log}\;x_{i}\right]x_{i}^{\alpha }e^{-\delta x^{\alpha }}{\left[A(x_{i})\right]}^{\theta -1}}{\text{sin}(\frac{\pi }{2}\{1-{\left[A(x_{i})\right]}^{\theta }\})}\\ & +\frac{k}{\alpha }+\sum_{i=1}^{k}\;\text{log}\;x_{i}-\delta \sum_{i=1}^{k}\left[\text{log}\;x_{i}\right]x_{i}^{\alpha }\\ & +(\theta -1)\sum_{i=1}^{k}\frac{\left[\text{log}\;x_{i}\right]x_{i}^{\alpha }e^{-\delta x^{\alpha }}}{\left[A(x_{i})\right]}, \end{array}$$

$$\begin{array}{cc} \frac{\partial }{\partial \delta }\xi (x_{1},x_{2},....,x_{k}|\theta ,\zeta ) & =\frac{\theta \pi }{2}\sum_{i=1}^{k}\text{sin}(\frac{\pi }{2}\{1-{\left[A(x_{i})\right]}^{\theta }\})x_{i}^{\alpha }e^{-\delta x_{i}^{\alpha }}{\left[A(x_{i})\right]}^{\theta -1}\\ & -\frac{\theta \pi }{2}\sum_{i=1}^{k}\frac{\text{cos}(\frac{\pi }{2}\{1-{\left[A(x_{i})\right]}^{\theta }\})x_{i}^{\alpha }e^{-\delta x_{i}^{\alpha }}{\left[A(x_{i})\right]}^{\theta -1}}{\text{sin}(\frac{\pi }{2}\{1-{\left[A(x_{i})\right]}^{\theta }\})}\\ & +\frac{k}{\delta }-\sum_{i=1}^{k}x_{i}^{\alpha }+(\theta -1)\sum_{i=1}^{k}\frac{x_{i}^{\alpha }e^{-\delta x_{i}^{\alpha }}}{\left[A(x_{i})\right]}, \end{array}$$

and

$$\begin{array}{cc} \frac{\partial }{\partial \theta }\xi (x_{1},x_{2},....,x_{k}|\theta ,\zeta ) & =\frac{\pi }{2}\sum_{i=1}^{k}\text{sin}(\frac{\pi }{2}\{1-{\left[A(x_{i})\right]}^{\theta }\})(\text{log}\;\left[A(x_{i})\right]){\left[A(x_{i})\right]}^{\theta }\\ & -\frac{\pi }{2}\sum_{i=1}^{k}\frac{\text{cos}(\frac{\pi }{2}\{1-{\left[A(x_{i})\right]}^{\theta }\})(\text{log}\;\left[A(x_{i})\right]){\left[A(x_{i})\right]}^{\theta }}{\text{sin}(\frac{\pi }{2}\{1-{\left[A(x_{i})\right]}^{\theta }\})}\\ & +\frac{k}{\theta }+\sum_{i=1}^{k}\;\text{log}\;[A(x_{i})], \end{array}$$

where $A(x_{i})=1-e^{-\delta x_{i}^{\alpha }}.$

Solving $\frac{\partial }{\partial \theta }\xi (x_{1},x_{2},....,x_{k}|\theta ,\zeta )$ and $\frac{\partial }{\partial \zeta }\xi (x_{1},x_{2},....,x_{k}|\theta ,\zeta ),$ we get the MLEs ${\hat{\theta }}_{MLE}$ and ${\hat{\zeta }}_{MLE}$ of the parameters *θ* and ***ζ*** respectively.

### 4.2 Simulation

Here, we demonstrate the performances of the MLEs $\left({\hat{\theta }}_{MLE},{\hat{\zeta }}_{MLE}\right)$ obtained in subsection 4.1. The demonstration of ${\hat{\theta }}_{MLE}$ and ${\hat{\zeta }}_{MLE}$ is done by considering random samples (RSs) of size *k* = 25, 50, 75, 100, 150, 200, 300, 400, 500 from the PDF of the TICE-Weibull model. The RSs are selected for two sets of *θ* and ***ζ***. The values of *θ* and ***ζ*** for these two sets are (i) *α* = 0.7, *δ* = 1.0, *θ* = 1.3, and (ii) *α* = 1.1, *δ* = 1.0, *θ* = 0.9.

For judging the behaviour of ${\hat{\theta }}_{MLE}$ and ${\hat{\zeta }}_{MLE}$, two statistical criteria (SC) are selected. These SC are given by (i) Biase, and (ii) mean square error (MLEs). The values of Bias and MSE are, respectively, calculated as

$$\begin{array}{c} Bias({\hat{\theta }}_{MLE})=\sum_{i=1}^{k}({\hat{\theta }}_{MLE}-\theta ), \end{array}$$

and

$$\begin{array}{c} MSE({\hat{\theta }}_{MLE})=\sum_{i=1}^{k}{\left({\hat{\theta }}_{MLE}-\theta \right)}^{2}. \end{array}$$

The above formulas are also implemented to obtain the values of Bias and MSE for the parameter vector ${\hat{\zeta }}_{MLE}.$

**Fig 2:**
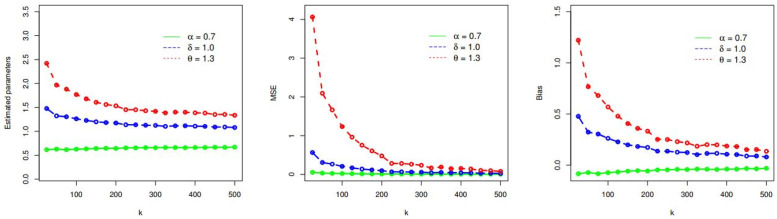
Visual presentation of the SRs of the TICE-Weibull model for *α* = 0.7, *δ* = 1.0, and *θ* = 1.3.

**Fig 3:**
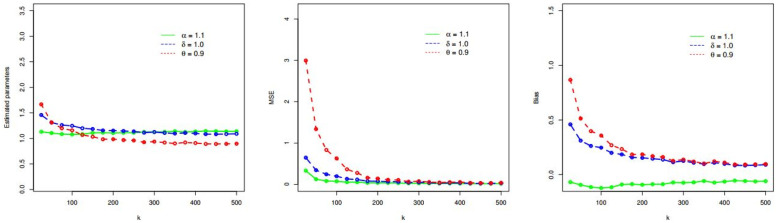
Visual presentation of the SRs of the TICE-Weibull model for *α* = 1.1, *δ* = 1.0, and *θ* = 0.9.

Corresponding to *α* = 0.7, *δ* = 1.0, *θ* = 1.3, the simulation results (SRs) are provided in Table 1 and presented graphically in Fig 2. Whereas, the SRs for *α* = 1.1, *δ* = 1.0, *θ* = 0.9 are presented in Table 2 and illustrated visually in Fig 3.

**Table 1:** The SRs of the TICE-Weibull model for *α* = 0.7, *δ* = 1.0, and *θ* = 1.3.

*n*	Parameters	MLEs	MSEs	Biases
25	*α*	0.6163352	0.05348795	-0.08366480
	*δ*	1.4773880	0.56458336	0.47738847
	*θ*	2.4209520	4.06299541	1.22095210
50	*α*	0.6280873	0.03288026	-0.07191270
	*δ*	1.3227470	0.30709121	0.32274704
	*θ*	1.9668570	2.09286722	0.76685710
75	*α*	0.6163906	0.02576817	-0.08360938
	*δ*	1.3044250	0.26543855	0.30442490
	*θ*	1.8808830	1.66363703	0.68088300
100	*α*	0.6269597	0.02208406	-0.07304032
	*δ*	1.2620040	0.20765034	0.26200382
	*θ*	1.7690360	1.23267780	0.56903590
150	*α*	0.6414660	0.01469801	-0.05853403
	*δ*	1.1989560	0.13788231	0.19895647
	*θ*	1.6069720	0.75371875	0.40697200
200	*α*	0.6428182	0.01186762	-0.05718179
	*δ*	1.1735110	0.09733192	0.17351075
	*θ*	1.5322040	0.47604446	0.33220420
300	*α*	0.6572279	0.00755858	-0.04277213
	*δ*	1.1233680	0.05712771	0.12336768
	*θ*	1.4173170	0.23569088	0.21731690
400	*α*	0.6618411	0.00629177	-0.03815893
	*δ*	1.1071790	0.04201144	0.10717940
	*θ*	1.3866370	0.15295632	0.18663730
500	*α*	0.6698825	0.00406900	-0.03011749
	*δ*	1.0800530	0.02503804	0.08005275
	*θ*	1.3355830	0.07803565	0.13558280

**Table 2:** The SRs of the TICE-Weibull model for *α* = 1.1, *δ* = 1.0, and *θ* = 0.9.

*n*	Parameters	MLEs	MSEs	Biases
25	*α*	1.1307240	0.33233716	-0.06927622
	*δ*	1.4595880	0.64708171	0.45958755
	*θ*	1.6669039	2.99543369	0.86690386
50	*α*	1.1056530	0.12507525	-0.09434740
	*δ*	1.3110040	0.34385483	0.31100391
	*θ*	1.3133591	1.33744014	0.51335910
75	*α*	1.0852750	0.08211869	-0.11472490
	*δ*	1.2616420	0.24417986	0.26164165
	*θ*	1.1976577	0.83304423	0.39765770
100	*α*	1.0773800	0.07529763	-0.12261993
	*δ*	1.2464940	0.19645634	0.24649405
	*θ*	1.1574794	0.62799457	0.35747939
150	*α*	1.1090990	0.05457913	-0.09090083
	*δ*	1.1842900	0.11565749	0.18429035
	*θ*	1.0336728	0.27753199	0.23367282
200	*α*	1.1066370	0.03844786	-0.09336277
	*δ*	1.1530400	0.07563832	0.15303957
	*θ*	0.9844707	0.14110588	0.18447074
300	*α*	1.1253090	0.02756519	-0.07469108
	*δ*	1.1250840	0.04987172	0.12508410
	*θ*	0.9372050	0.07646262	0.13720498
400	*α*	1.1357700	0.01935284	-0.06422967
	*δ*	1.0983210	0.03617090	0.09832051
	*θ*	0.9095661	0.05527063	0.10956606
500	*α*	1.1393110	0.01748218	-0.06068903
	*δ*	1.0902410	0.02871474	0.09024133
	*θ*	0.8965754	0.03912552	0.09657542

## 5 Data modeling

This section is concerned with an illustration of the usefulness of the TICE-Weibull distribution for modeling the lifetime scenarios of electronic components. Both the data sets are taken from the domain of the engineering sectors to illustrate the fitness of the TICE-Weibull model in comparison (competition) to other selected models, namely, Weibull, new generalized exponential-Weibull (NGE-Weibull), and new extended exponential Weibull (NEE-Weibull) distributions. The SFs of the competing distributions are

- Weibull
  $$\begin{array}{c} S(x;\zeta )=e^{-\delta x^{\alpha }},\;\;\;\;\;\;\;\;x>0,\alpha ,\delta >0. \end{array}$$
- NGE-Weibull
  $$\begin{array}{c} S(x;\kappa ,\varphi ,\zeta )={\left(\frac{\varphi e^{-\delta x^{\alpha }}}{\varphi +1-e^{-\delta x^{\alpha }}}\right)}^{\kappa },\;\;\;\;\;\;\;\;\;x>0,\alpha ,\delta ,\kappa ,\varphi >0. \end{array}$$
- NEE-Weibull
  $$\begin{array}{c} S(x;\varphi ,\zeta )=(\frac{\varphi e^{-\delta x^{\alpha }}}{\varphi +1-e^{-\delta x^{\alpha }}}),\;\;\;\;\;\;\;\;\;x>0,\alpha ,\delta ,\varphi >0. \end{array}$$

The Cramer-Von-Messes (denoted by W*) test, Anderson Darling (denoted by A*) test, and Kolmogorov Simonrove (denoted by KS*) test *P* value are taken to compare the TICE-Weibull and other selected models (candidate models).

- The W* test is one of the most widely used measures to compare the fitting capability of the estimated models. For the underlined data, a model with the smaller value of W* test has the best fit. The W* test is defined as
  $$\begin{array}{c} \sum_{i=1}^{k}{\left[\frac{2i-1}{2k}-G(x_{i})\right]}^{2}+\frac{1}{12k}, \end{array}$$
  where *k* and *x*_*i*_ denote the sample size and *i*^*th*^ observation of the underlined data, respectively.
- The A* test is also used as a comparative measure for the competing models. This test is computed as
  $$\begin{array}{c} -\frac{1}{k}\sum_{i=1}^{k}[\text{log}\{1-G(x_{k-i+1})\}+\text{log}G(x_{i})](2i-1)-k. \end{array}$$
- Let *G*_*k*_(*x*) and $\hat{G}(x)$, respectively, represent the estimated CDF of the selected model (given model) and empirical CDF of the sample values. Then, the KS* is computed by
  $$\begin{array}{c} sup_{x}[G_{k}(x)-\hat{G}(x)]. \end{array}$$

### 5.1 Data 1

**Fig 4:**
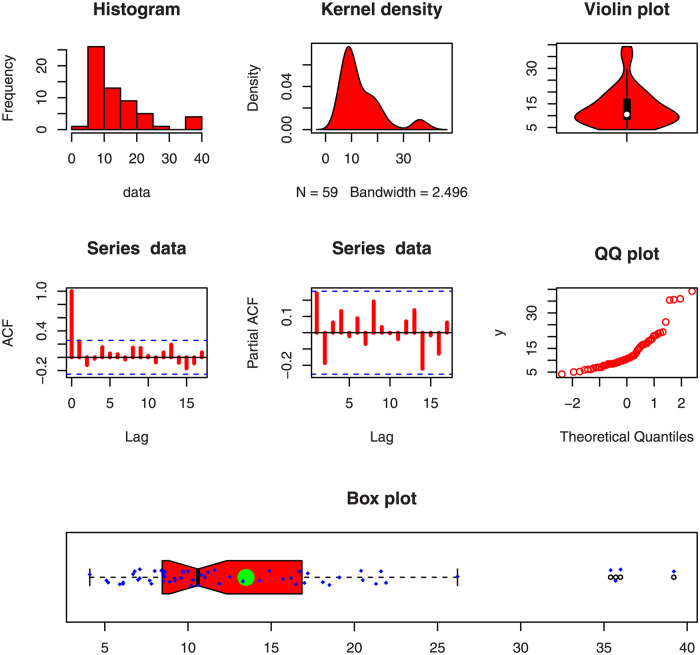
Visual illustration of the first data set through basic plots.

This data represents the lifetimes of fifty-nine electronic devices. Corresponding to the first data set, the key values are provided in Table 3. Some descriptive plots of the Data 1 are presented in Fig 4.

**Table 3:** The first data set with corresponding summary statistics.

Min.	Max.	$\bar{x}$	*Var*(*x*)	Median	*SD*(*x*)
4.100	39.200	13.490	64.826	10.600	8.051
*Q*_1_	*k*	*Q*_3_	Skewness	Kurtosis	Range
8.450	59	16.85	1.608	5.256	35.100

**Fig 5:**
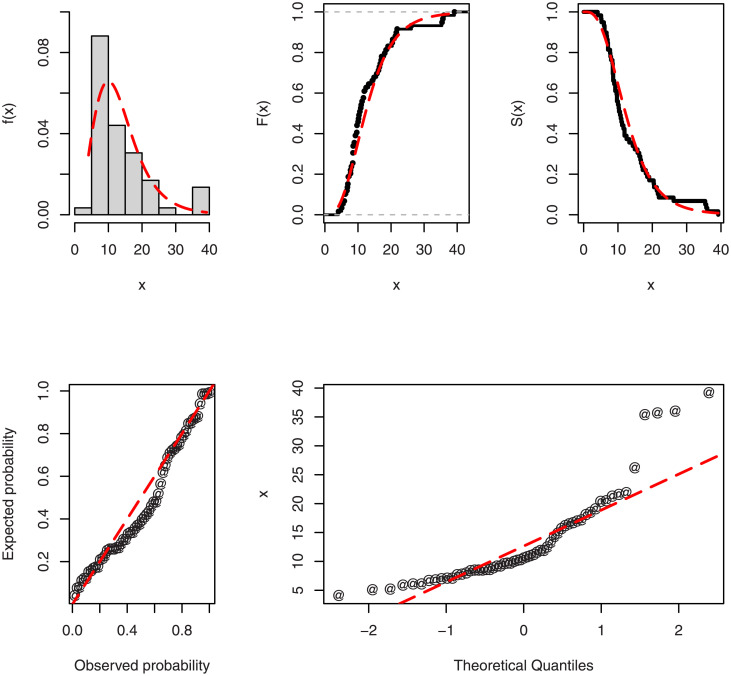
For the first data set, the estimated plots of the basic functions of the TICE-Weibull model.

Tables 4 and 5, respectively, display the MLEs and values of the selection criteria (i.e., W*, A*, KS*, and *P* value) of TICE-Weibull and other candidate models. Since the values of the W*, A*, and KS* of the TICE-Weibull model are the smallest among those other competing models, therefore, the TICE-Weibull model appears to be the best model. The fitted density (PDF), probability-probability (PP), CDF, quantile-quantile (QQ), and SF plots of the TICE-Weibull model to Data 1 are displayed in Fig 5. From the given plots in Fig 5, it can be seen that the TICE-Weibull model closely fits Data 1.

**Table 4:** For the first data set, the values of ${\hat{\alpha }}_{MLE},{\hat{\delta }}_{MLE},{\hat{\theta }}_{MLE},{\hat{\kappa }}_{MLE}$, and ${\hat{\phi }}_{MLE}$ of the competing distributions.

Models	${\hat{\alpha }}_{MLE}$	${\hat{\delta }}_{MLE}$	${\hat{\theta }}_{MLE}$	${\hat{\kappa }}_{MLE}$	${\hat{\phi }}_{MLE}$
TICE-Weibull	0.60819	0.53160	9.33344	-	-
Weibull	1.86673	0.00621	-	-	-
NGE-Weibull	2.00500	0.00598	-	0.595360	2.32846
NEE-Weibull	2.01242	0.00325	-	-	3422712

**Table 5:** For the first data set, the values of SC of the competing distributions.

Models	W*	A*	KS*	*P* value
TICE-Weibull	0.17588	1.04932	0.13045	0.26790
Weibull	0.29636	1.89188	0.14122	0.18990
NGE-Weibull	0.27951	1.80245	0.13631	0.22290
NEE-Weibull	0.26909	1.72379	0.14537	0.16510

### 5.2 Data 2

**Fig 6:**
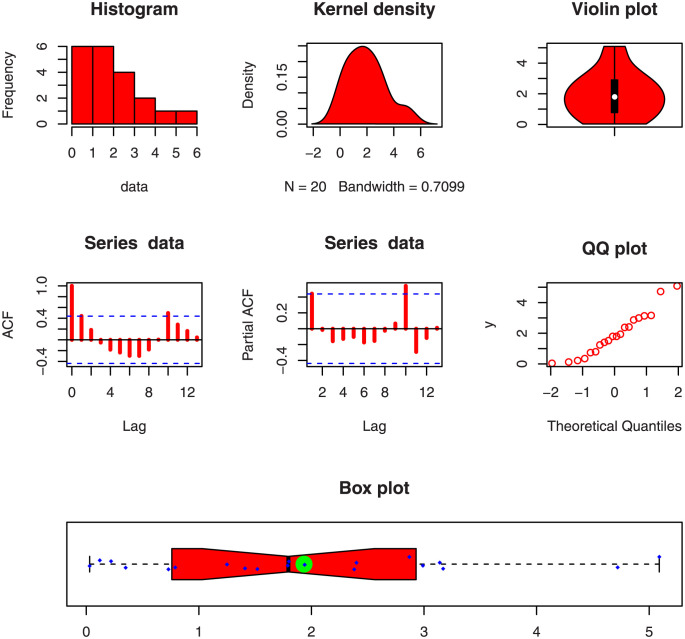
Visual illustration of the second data set through basic plots.

Data 2 denote the lifetimes of twenty electronic components. The observations of Data 2 with the associated summary values are displayed in Table 6. Besides the key measures, certain descriptive plots of Data 2 are also obtained; see Fig 6.

**Table 6:** The second data set with corresponding summary statistics.

Min.	Max.	$\bar{x}$	*Var*(*x*)	Median	*SD*(*x*)
0.030	5.090	1.935	2.062	1.795	1.436
*Q*_1_	*k*	*Q*_3_	Skewness	Kurtosis	Range
0.775	20	2.900	0.602	2.720	5.060

Based on the statistical analysis of Data 2, the MLEs and values of the selection criteria of TICE-Weibull and other candidate models are displayed in Tables 7 and 8, respectively. Based on Table 8, we can see that the TICE-Weibull model again turns out to be the best suitable distribution.

**Table 7:** For the second data set, the values of ${\hat{\alpha }}_{MLE},{\hat{\delta }}_{MLE},{\hat{\theta }}_{MLE},{\hat{\kappa }}_{MLE}$, and ${\hat{\phi }}_{MLE}$ of the competing distributions.

Models	${\hat{\alpha }}_{MLE}$	${\hat{\delta }}_{MLE}$	${\hat{\theta }}_{MLE}$	${\hat{\kappa }}_{MLE}$	${\hat{\phi }}_{MLE}$
TICE-Weibull	2.25489	0.02901	0.36511	-	-
Weibull	1.19651	0.42700	-	-	-
NGE-Weibull	1.18330	0.04298	-	7.78103	3.77559
NEE-Weibull	1.20661	0.41386	-	-	9.03016

**Table 8:** For the second data set, the values of SC of the competing distributions.

Models	W*	A*	KS*	*P* value
TICE-Weibull	0.03202	0.20795	0.10292	0.96930
Weibull	0.07113	0.41836	0.12746	0.86130
NGE-Weibull	0.07535	0.44216	0.12127	0.89650
NEE-Weibull	0.07427	0.43557	0.14402	0.74890

**Fig 7:**
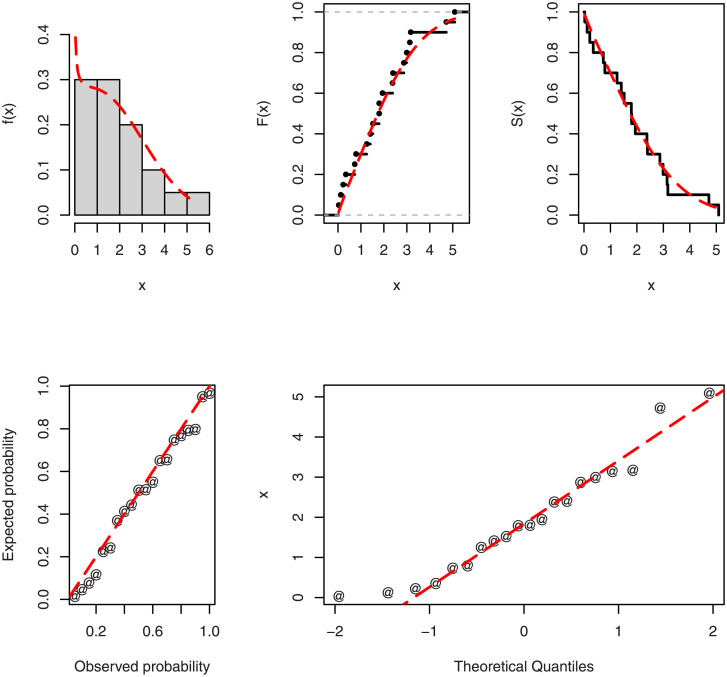
For the second data set, the estimated plots of the basic functions of the TICE-Weibull model.

Some fitted plots of the TICE-Weibull model are shown in Fig 7. From these plots (see Fig 7), it can be observed that the estimated CDF and PDF of TICE-Weibull distribution closely follow the empirical CDF and the pattern of the histogram on Data 2, respectively. This behavior of the TICE-Weibull has also been confirmed by obtaining the PP, empirical SF, and QQ plots.

## 6 Attribute control chart

There are assorted valuable control charts to maintain the manufacturing process under control, comprising the lie between the lower control limit (LCL) and upper control limit (UCL). In the statistical quality control process, there are two renowned types of control charts, namely, the control chart for attributes and the control chart for variables. The variables control chart provides good information considering the process and comprises minimum sample sizes; since it applies quantitative data. The use of an attribute control chart is more flexible as compared to the variables chart due to its easiness of computation. The various attribute control chart are renowned in the literature such as the *np* chart, *u* chart, and the *c* chart.

The attribute control chart for time truncated life test for various distributions has been studied by various authors, please refer to Haq and Al-Omari [19], Quinino et al. [20], Rao and Al-Omari [21] and Adeoti and Rao [22]. By exploring the literature there is no work on an attribute control chart based on the TICE-Weibull distribution. In this paper, a new attribute control chart based on a truncated life test is projected using the TICE-Weibull distribution.

### 6.1 The proposed control chart

We project a new *np* control chart founded on time-truncated life testing as developed by Aslam and Jun [23]:

- Step 1
  Chose a simple random sample of size *n* from the submitted lot and examine them. The number of failures dented by *φ* is obtained before the experiment time *x*_0_ = *τξ*_0_, where *ξ*_0_ is the quality consideration under the condition that the process is in-control and *τ* is a multiplier constant.
- Step 2
  Declare that the process as out-of-control when *φ* > *UCL* and *φ* < *LCL* otherwise the process is in-control if *LCL* < *φ* < *UCL*.

The percentile of the T-Weibull distribution is

$$\begin{array}{c} \varsigma_{q}={\left(-\frac{1}{\delta }\text{log}[1-{\left\{1-\frac{2}{\pi }{\text{cos}}^{-1}(\text{log}[q(e-1)+1])\right\}}^{1/\theta }]\right)}^{1/\alpha }. \end{array}$$ (15)

Let

$$\begin{array}{c} \delta =\frac{\varsigma_{q}}{x_{q}^{\alpha }}\;, \end{array}$$

where

$$\begin{array}{c} \varsigma_{q}=-\text{log}[1-{\left\{1-\frac{2}{\pi }{\text{cos}}^{-1}(\text{log}[q(e-1)+1])\right\}}^{1/\theta }]. \end{array}$$

Using the binomial distribution of defective products with parameters *p*_0_ and *n* the proposed chart limits are, respectively, obtained as

$$\begin{array}{c} UCL=np_{0}+L\sqrt{np_{0}-(1-p_{0})}, \end{array}$$

and

$$\begin{array}{c} LCL=max\{0,np_{0}-L\sqrt{np_{0}-(1-p_{0})}\}, \end{array}$$

where *p*_0_ is the probability of failed article before the testing time *x*_0_ when the process considered as in-control, and *L* is the chart coefficient to be obtained. On the other hand, we can say the process is in-control once *ξ* = *ξ*_0_(*i*.*e*., *α* = *α*_0_, *θ* = *θ*_0_, *δ* = *δ*_0_).

Let us consider that the experiment time is *x*_0_ as multiple of termination ratio $\varsigma_{q}$ and specified percentile life *x*_*q*0_, i.e., $\varsigma_{q}x_{q0}$ in time-truncated lifetime experimentation. After simplification, the probability of failure is written as

$$\begin{array}{c} p=\frac{e^{\text{cos}(\frac{2}{\pi }{\left[1-(1-e^{-\varsigma_{q}{\left(\frac{\tau }{x_{q}/x_{q0}}\right)}^{\alpha }})\right]}^{\theta })}-1}{e-1}. \end{array}$$ (16)

When the process is in-control, then the percentile ratio $\frac{x_{q}}{x_{q0}}=1.$ Therefore, Eq (16) will be reduced to

$$\begin{array}{c} p_{0}=\frac{e^{\text{cos}(\frac{2}{\pi }{\left[1-(1-e^{-\varsigma_{q}{\left(\tau \right)}^{\alpha_{0}}})\right]}^{\theta_{0}})}-1}{e-1}. \end{array}$$ (17)

Now, we consider the percentile ratio *x*_*q*_/*x*_*q*0_ = *c* = 1.0, 1.05, 1.10, …, 4.0 Then, the probability in Eq (17) becomes

$$\begin{array}{c} p_{1}=\frac{e^{\text{cos}(\frac{2}{\pi }{\left[1-(1-e^{-\varsigma_{q}{\left(\frac{\tau }{c}\right)}^{\alpha_{0}}})\right]}^{\theta_{0}})}-1}{e-1}. \end{array}$$

Let $\bar{\varphi }$ denote the average of failures for the subgroups over the sample. If the value of *p*_0_ is not known, the chart limits for realistic purposes can be used in the following expressions

$$\begin{array}{c} UCL=\bar{\varphi }+k\sqrt{\bar{\varphi }(1-\bar{\varphi }/n)}, \end{array}$$

and

$$\begin{array}{c} LCL=max\{0,\bar{\varphi }-k\sqrt{\bar{\varphi }(1-\bar{\varphi }/n)}\}. \end{array}$$

The chance of declaring as the process is in-control for the developed control chart is given by

$$\begin{array}{c} P_{IC}^{0}=P\left\{LCL\leq D\leq UCL|p_{0}\right\}=\sum_{k=\lfloor UCL\rfloor +1}^{\left\lfloor UCL\right\rfloor }\left(\begin{array}{c} n\\ k \end{array}\right)p_{0}^{k}{\left(1-p_{0}^{k}\right)}^{n-k}. \end{array}$$

The accomplishment of the developed control chart can be examined by its average control length (ARL) and when the process as in-control state it is expressed as follows:

$$\begin{array}{c} ARL_{0}=\frac{1}{1-P_{IC}^{0}}. \end{array}$$

To investigate the performance of the proposed control chart, the study of out-of-control is needed to investigate. When the process is out-of-control, assume that *p*_1_ as the probability of an unsuccessful item earlier than the experiment time *x*_0_ Hence, the chance that the process is evident to be in control, whilst the declared time ratio is changed to *c* is given by

$$\begin{array}{c} P_{IC}^{1}=P\left\{LCL\leq D\leq UCL|p_{1}\right\}=\sum_{k=\lfloor UCL\rfloor +1}^{\left\lfloor UCL\right\rfloor }\left(\begin{array}{c} n\\ k \end{array}\right)p_{1}^{k}{\left(1-p_{1}^{k}\right)}^{n-k}. \end{array}$$ (18)

The following step-by-step procedure can be used to acquire the tables of the developed control chart

- Find out the ARL value, say *r*_0_, and known parametric values *α* = *α*_0_, *θ* = *θ*_0_, *δ* = *δ*_0_, *α*_0_.
- Determine the chart constants *L*, *τ*, and *n* such that the *ARL*_0_ value is almost equal to *r*_0_, i.e., *ARL*_0_ > *r*_0_.
- Subsequent to receiving the values in the above step, determine the *ARL*_1_ according to shift constant *c* based on Eq (18).

We determined the control chart parameters and *ARL*_1_ for various values of *α* = *α*_0_, *θ* = *θ*_0_, *r*_0_, and *n*, given in Tables 9–12 for shift values.

**Table 9:** ARL values of the proposed chart for *θ* = 1.3, *α* = 0.7, and *n* = 20.

*r*_0_	200	250	300	370	500
*L*	2.795	2.956	2.938	3.029	3.141
*a*	0.756	0.666	1.082	0.968	1.334
*c*	ARL1	ARL1	ARL1	ARL1	ARL1
1.00	200.18	250.24	300.28	370.23	500.47
1.05	161.24	273.10	176.20	261.49	307.36
1.10	113.09	231.44	103.23	162.93	170.51
1.15	77.60	172.42	63.63	102.15	97.89
1.20	54.41	124.32	41.48	66.74	59.68
1.25	39.39	90.48	28.45	45.62	38.58
1.30	29.45	67.35	20.40	32.51	26.27
1.35	22.68	51.41	15.20	24.03	18.72
1.40	17.93	40.20	11.71	18.34	13.87
1.45	14.50	32.12	9.28	14.40	10.64
1.50	11.97	26.18	7.55	11.59	8.41
1.55	10.06	21.70	6.28	9.53	6.82
1.60	8.59	18.28	5.32	7.98	5.65
1.65	7.44	15.61	4.59	6.80	4.79
1.70	6.52	13.49	4.01	5.88	4.12
1.75	5.78	11.80	3.56	5.16	3.61
1.80	5.18	10.42	3.19	4.57	3.20
1.85	4.68	9.28	2.90	4.10	2.87
1.90	4.26	8.34	2.65	3.71	2.61
1.95	3.91	7.55	2.45	3.38	2.39
2.00	3.61	6.88	2.27	3.11	2.21
2.50	2.10	3.54	1.45	1.79	1.38
3.00	1.58	2.42	1.20	1.38	1.15
3.50	1.35	1.92	1.10	1.21	1.07
4.00	1.23	1.64	1.05	1.12	1.03

**Table 10:** ARL values of the proposed chart for *θ* = 1.3, *α* = 1.1, and *n* = 20.

*r*_0_	200	250	300	370	500
*L*	2.888	2.954	4.124	3.064	3.141
*a*	1.154	1.257	0.571	1.166	0.647
*c*	ARL1	ARL1	ARL1	ARL1	ARL1
1.00	200.30	250.98	300.74	370.42	501.59
1.05	150.40	160.52	213.59	223.32	487.87
1.10	93.81	92.18	154.49	112.87	394.43
1.15	57.81	54.46	114.85	66.62	296.00
1.20	37.12	34.08	87.76	41.91	219.59
1.25	25.05	22.59	68.77	27.92	165.04
1.30	17.71	15.76	55.11	19.54	126.59
1.35	13.05	11.50	45.03	14.27	99.19
1.40	9.97	8.72	37.45	10.82	79.30
1.45	7.86	6.84	31.62	8.47	64.56
1.50	6.36	5.52	27.06	6.82	53.43
1.55	5.28	4.57	23.44	5.62	44.87
1.60	4.47	3.87	20.52	4.74	38.18
1.65	3.85	3.34	18.15	4.06	32.87
1.70	3.38	2.93	16.18	3.55	28.61
1.75	3.00	2.61	14.54	3.14	25.13
1.80	2.70	2.36	13.16	2.81	22.27
1.85	2.46	2.15	11.99	2.55	19.89
1.90	2.26	1.99	10.99	2.34	17.89
1.95	2.09	1.85	10.12	2.16	16.20
2.00	1.95	1.73	9.37	2.01	14.75
2.50	1.30	1.21	5.27	1.32	7.36
3.00	1.12	1.07	3.69	1.13	4.79
3.50	1.05	1.03	2.91	1.05	3.59
4.00	1.02	1.01	2.45	1.03	2.92

**Table 11:** ARL values of the proposed chart for *θ* = 1.3, *α* = 0.7, and *n* = 40.

*r*_0_	200	250	300	370	500
*L*	2.917	2.917	3.047	3.113	3.140
*a*	1.403	0.984	1.273	1.454	1.461
*c*	ARL1	ARL1	ARL1	ARL1	ARL1
1.00	200.65	250.67	300.49	370.20	500.93
1.05	103.79	219.69	164.21	171.50	189.44
1.10	48.05	120.55	75.35	73.80	78.68
1.15	24.45	63.37	37.51	35.50	37.43
1.20	13.86	35.70	20.73	19.17	20.07
1.25	8.63	21.74	12.57	11.44	11.91
1.30	5.82	14.19	8.25	7.43	7.69
1.35	4.20	9.83	5.79	5.18	5.34
1.40	3.21	7.17	4.30	3.84	3.94
1.45	2.57	5.46	3.35	3.00	3.06
1.50	2.14	4.32	2.72	2.44	2.48
1.55	1.84	3.53	2.28	2.06	2.09
1.60	1.63	2.96	1.97	1.79	1.81
1.65	1.47	2.55	1.74	1.60	1.61
1.70	1.36	2.24	1.58	1.45	1.47
1.75	1.28	2.00	1.45	1.35	1.36
1.80	1.21	1.81	1.35	1.27	1.28
1.85	1.16	1.67	1.28	1.21	1.21
1.90	1.12	1.55	1.22	1.16	1.16
1.95	1.10	1.46	1.18	1.12	1.13
2.00	1.07	1.38	1.14	1.10	1.10
2.50	1.01	1.07	1.01	1.01	1.01
3.00	1.00	1.02	1.00	1.00	1.00
3.50	1.00	1.00	1.00	1.00	1.00
4.00	1.00	1.00	1.00	1.00	1.00

**Table 12:** ARL values of the proposed chart for *θ* = 1.3, *α* = 1.1, and *n* = 40.

*r*_0_	200	250	300	370	500
*L*	2.921	2.897	3.05	3.113	3.098
*a*	1.241	0.962	0.846	1.269	0.707
*c*	ARL1	ARL1	ARL1	ARL1	ARL1
1.00	200.41	250.10	300.16	370.31	500.06
1.05	104.61	149.69	268.37	171.59	478.86
1.10	48.42	76.42	158.08	73.84	308.23
1.15	24.62	41.31	88.16	35.52	182.42
1.20	13.94	24.28	51.79	19.18	111.25
1.25	8.67	15.40	32.45	11.44	71.36
1.30	5.85	10.43	21.57	7.43	48.07
1.35	4.22	7.47	15.10	5.19	33.83
1.40	3.22	5.61	11.05	3.84	24.73
1.45	2.57	4.39	8.40	3.00	18.68
1.50	2.14	3.56	6.61	2.44	14.53
1.55	1.84	2.97	5.34	2.06	11.60
1.60	1.63	2.54	4.43	1.79	9.46
1.65	1.48	2.22	3.76	1.60	7.87
1.70	1.36	1.98	3.25	1.45	6.67
1.75	1.28	1.80	2.86	1.35	5.74
1.80	1.21	1.65	2.55	1.27	5.00
1.85	1.16	1.54	2.30	1.21	4.42
1.90	1.13	1.44	2.10	1.16	3.94
1.95	1.10	1.37	1.94	1.12	3.56
2.00	1.07	1.31	1.81	1.10	3.24
2.50	1.01	1.06	1.22	1.01	1.76
3.00	1.00	1.01	1.07	1.00	1.34
3.50	3.50	1.00	1.03	1.00	1.17
4.00	4.00	1.00	1.01	1.00	1.09

Based on computed tables, we observed the following conclusions

- It is observed that *ARL*_1_ value in decreasing tendency as the shift value *c* increases.
- From Tables 9 and 10, it is evident that for fixed *θ* value ARL1 values are decreases with the increase of parameter *α*.
- Based on Tables 9–12, it is reasonable that when the sample size *n* increases from 20 to 40, the *ARL*_1_ values are shows decreasing tendency.

### 6.2 Illustration of the suggested control chart

The demonstration of the developed control chart is as follows: let us assume that industrial output persists the TICE-Weibull distribution with parameters *α* = 0.7, *θ* = 1.3. Suppose the average target lifetime of the product is *ξ*_0_ = 1000 hours and *r*_0_ = 370. Using the Eq (17) the value of *p*_0_ is 0.48866. Also, from Table 13, the chart parameters are *n* = 20, *τ* = 0.968, *L* = 3.029, *LCL* = 3, and *UCL* = 16. Hence, the experiment time *x*_0_ is 968 hours. Thus, the proposed control chart is carried out in the following steps:

- Step I
  Draw a simple random sample of size 20 from every subgroup and put them for the life testing assessment during 968 hours. Determine the number of failed units say *D* for the duration of the experiment time.
- Step II
  Pronounce the production process as under control if 3 ≤ *D* ≤ 16; else, the production process can be regarded as out-of-control.

**Table 13:** ARL values of the proposed chart for $\hat{\theta }=0.3651,\hat{\alpha }=2.2549,$ and *n* = 20.

*r*_0_	200	250	300	370	500
*L*	2.881	2.956	2.939	3.06	3.66
*a*	1.192	1.314	1.064	1.094	0.536
*c*	ARL1	ARL1	ARL1	ARL1	ARL1
1.00	200.95	250.15	301.04	371.18	500.33
1.05	187.36	213.66	246.30	317.21	477.06
1.10	165.52	175.65	200.40	263.88	446.56
1.15	141.70	142.32	163.75	217.51	413.12
1.20	119.56	115.26	134.98	179.48	379.60
1.25	100.53	93.98	112.44	149.04	347.67
1.30	84.76	77.39	94.70	124.85	318.16
1.35	71.90	64.45	80.61	105.59	291.37
1.40	61.47	54.28	69.31	90.16	267.30
1.45	52.98	46.22	60.16	77.70	225.80
1.50	46.04	39.75	52.67	67.56	206.64
1.55	40.33	34.52	46.48	59.21	189.56
1.60	35.59	30.23	41.32	52.30	164.34
1.65	31.63	26.69	36.97	46.51	150.74
1.70	28.30	23.75	33.29	41.63	128.56
1.75	25.48	21.27	30.15	37.48	107.63
1.80	23.06	19.17	27.44	33.94	97.79
1.85	20.99	17.37	25.10	30.88	88.91
1.90	19.19	15.83	23.06	28.24	60.87
1.95	17.63	14.50	21.28	25.93	43.57
2.00	16.27	13.34	19.71	23.91	26.92
2.50	8.67	7.01	10.77	12.61	13.87
3.00	5.71	4.61	7.15	8.17	10.66
3.50	4.24	3.45	5.32	5.97	8.50
4.00	3.41	2.79	4.26	4.71	5.78

### 6.3 An industrial application

**Fig 8:**
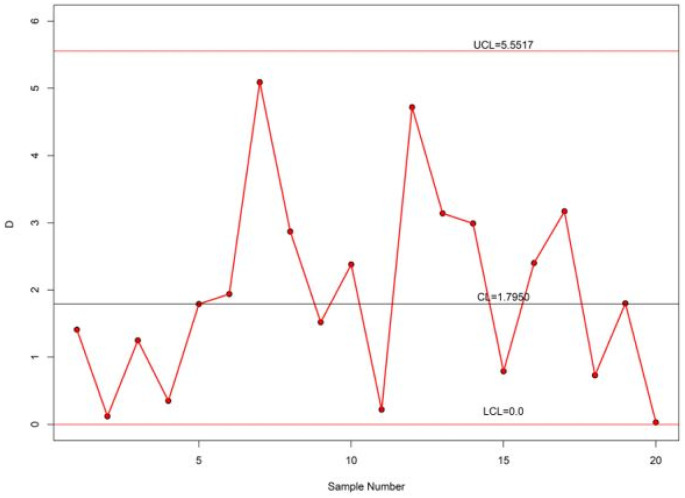
Proposed control chart for real data.

Using the second data set, the estimated parameters of the TICE-Weibull distribution are $\hat{\theta }=0.3651,\hat{\alpha }=2.2549,$ and $\hat{\delta }=0.02901.$ Table 13 provides the ARLs of the proposed control chart for the second data set. Here, $n=20,\hat{\theta }=0.3651,\hat{\alpha }=2.2549,\tau =1.064,$ and *r*_0_ = 300. The value of *p*_0_ is 0.5281 using Eq (17). The value of *ξ* by Eq (15) is obtained as 4.1835 for the duration of test *x*_0_ = *τξ*_0_ = (1.064)(4.1835) = 4.4512. The control limits of the proposed chart are *LCL* = 0 and *UCL* = 5.5517 for the parameter *L* = 2.939. The proposed control chart for the lifetimes of twenty electronic components data is depicted in Fig 8. From Fig 8, it is noticed that the proposed chart shows lifetimes of electronic components data are under control. Hence, the proposed chart is suitable to monitor the quality of the electronic components.

### 6.4 Comparison

The ARL values of the proposed control chart and the existing time truncated life testing attributed control charts for the Weibull distribution given in Adeoti and Rao (2021) are compared. The results of comparisons between the TICE-Weibull and Weibull distributions are displayed in Table 14. To compare the two types of control charts with respect to ARL values at different shift values. It is important to note that a chart having lesser out-of-control ARLs would be considered the better control chart. We noticed based on Table 14, the ARL values of the developed control chart have fever ARLs as compared with the control chart developed for the Weibull distribution. For instance, when *c* = 1.4, the *ARL*_1_ of the developed TICE-Weibull control chart for *n* = 20 is 18.34. Whereas, the *ARL*_1_ for the Weibull distribution is 13.51. Hence, we conclude that the proposed chart is speedy to find process changes as compared with the existing control chart established on the Weibull distribution.

**Table 14:** ARLs of attribute control charts for TICE-Weibull and Weibull distributions when *ARL*_0_ = 370 and *n* = 20.

*L*	3.029	2.8495
*τ*	0.968	0.6959
*c*	TICE-Weibull	Weibull
1.0	370.23	370.1
1.1	162.93	438.1
1.2	66.74	193.79
1.3	32.51	89.99
1.4	18.34	48.47
1.5	11.59	29.4
1.6	7.98	19.52
1.7	5.88	13.9
1.8	4.57	10.46
1.9	3.71	8.23
2.0	3.11	6.69

## 7 Final remarks

In this study, a new trigonometric version of Weibull distribution with the implementation of a trigonometric function was investigated. The TICE-Weibull model can be considered a useful updated version of the Weibull model. Certain statistical properties including shapes of PDF along with the IP, QF, and *m*^*th*^ moment have been studied. The MLEs of the parameters involved in the TICE-Weibull distribution are obtained. Two real datasets were considered for illustration of the TICE-Weibull distribution. Moreover, an attribute control chart is proposed for TICE-Weibull distribution. The proposed control chart coefficient and out-of-control ARLs for various sample sizes and parametric values are given in Tables 9–12. The results show that the proposed control chart shows its efficiency at different sample sizes as compared with the existing Weibull distribution. Finally, the proposed control chart was also demonstrated with the help of real data for industrial application. Based on our findings, it is observed that the TICE-Weibull distribution control chart is better than the Weibull distribution control chart for monitoring the lifetime of electronic components.

In the future, we are motivated to apply the proposed distribution to the dynamical systems via data-driven approaches such as the non-Gaussian system control. We are also intended to obtain the bivariate extension of the proposed model for analyzing the bivariate data sets. Furthermore, the proposed control chart can also be implemented to monitor other industrial processes like chemical, electrical or mechanical energy in the manufacturing of industrial products or pharmaceutical manufacturing processes using other models like repetitive, multiple dependent state sampling etc.
